# Performance Evaluation of a Novel Potentiometric Membrane Sensor for Determination of Atorvastatin in Pharmaceutical Preparations 

**Published:** 2013

**Authors:** Farhad Ahmadi, Nasim Asaadi

**Affiliations:** a *Drug Delivery Research Center, Faculty of Pharmacy, Kermanshah University of Medical Sciences, Kermanshah, Islamic Republic of Iran.*; b *Department of Medicinal Chemistry, Faculty of Pharmacy, Kermanshah University of Medical Sciences, Kermanshah, Islamic Republic of Iran. *

**Keywords:** Atorvastatin, PVC membrane, Potentiometric sensor, Analysis of pharmaceutical preparation

## Abstract

A novel potentiometric ion-selective PVC membrane sensor for analysis of atorvastatin (AT) in pharmaceutical preparations based on atorvastatin-(tetraphenyl borate), (AT-(TPB)2) as sensing element, tetraphenyl borate as additive and tris-2-ethyl-hexyl phosphate (TOP) as plasticizer solvent was prepared. The electrode shows a good Nernestian response over the concentration range of 0.09–5586 μg mL^-1^of AT with slope of 30.1±0.1 mV/decade and limit of detection0.056μg mL^-1^.The response time of sensor is fats (less than 10 sec) and could be used for about one month in the pH range of 4.5–8.0. The electrode exhibit good selectivity for the AT in the presence of large amount of co-drugs and inorganic cations. The method is precise and accurate with mean relative standard deviation of <2%.Atorvastatin is determined successfully in several tablets by the proposed membrane.

## Introduction

Atorvastatin, R-(R*,R*)]-2-(4-fluorophenyl)- *β,δ*-dihydroxy-5-(1-methylethyl)-3-phenyl- 4-[(phenylamino) carbonyl]-1H-pyrrole-1- heptanoic acid (pKa = 4.46, log P = 6.36), is a synthetic lipid-lowering agent (1, 2). Atorvastatin (AT) is an inhibitor of 3-hydroxy- 3-methylglutaryl-coenzyme A (HMG-CoA) reductase. This enzyme catalyzes the conversion of HMG-CoA to mevalonate, an early and rate-limiting step in cholesterol biosynthesis (3). It is applied for reducing of LDL-cholesterol, apolipoprotein B, and triglycerides and increasing of HDL-cholesterol in the treatment of hyperlipidaemias (4-7). Also, AT is indicated to diminish the risk of myocardial infarction (8), stroke (9) which decrease the risk for revascularization procedures (10) and angina (11). AT is rapidly absorbed after oral administration, however, due to pre-systematic clearance in the gastro-intestinal mucosa and metabolism in the liver, its absolute bioavailability is approximately 12%, and low plasma concentration is followed after taking the drug (5, 12). There are many reported methods for the determination of AT alone (13-20) or in combination with other drugs especially with amlodipine in pharmaceutical dosage forms or individually in biological fluids. Existing analytical methods for AT included: spectrophotometric methods (21, 22), HPLC-UV (23, 24), FT-Raman spectroscopy (25), automated enzyme inhibition assay method using radioactivity detection (26), UPLC (27) and also HPLC equipped with mass spectrometry detection (13-15). Novakora and coworkers, reported the HPLC methods for determination of AT in various fields of applications, including bio-analytical assays, pharmaceutical assays and environmental applications (28). Although the chromatographic methods are more sensitive and specific, but these devices requires sophisticated and expensive instruments, a specific column for each determination and special care with reagents before its injection into the chromatographic system (filtration, extraction and degassing) (23, 29-31). Furthermore, automated enzyme inhibition assay method need to long sample pretreatingtimes (26) and FT-Raman spectroscopy (25) has a small calibration range. Without all of these drawbacks and presenting high analytical ranges and reproducibility’s, minimal interference from associated and related species, direct application to turbid and colored drug solutions without any pre-treatment and low cost and easy operation of the potentiometric instrumentation, direct potentiometry using conventional ion selective electrodes (ISEs) is nowadays a highly desirable alternative for the analysis of pharmaceutical compounds (32-34). In the present work, a plastic membrane electrode is prepared based on incorporation of an ion pair complex of sodium tetraphenylborate (NaTPB) anion with AT in a PVC matrix plasticized with tris-2-ethyl-hexyl phosphate (TOP). The electrode was applied for analysis a range of AT concentrations in the region 0.089–5586 μg mL-1 using the Nernstain equation.

## Experimental

*Reagents and materials*

The pure form of atorvastatin (C_33_H_35_FN_2_O_5_, gfw=558.7) used in the present work was supplied by Bakhtar Bioshimi Pharmaceutical Company (Kermanshah, Iran). All chemicals used where of analytical or pharmaceutical grade and solutions were prepared in double distilled water. Dibutyl phthalate (DBP), dioctyl phthalate (DOP), tri-butyl phosphate (TBP), tris-2-ethyl-hexyl phosphate (TOP), 2-nitrophenyloctyl ether (2-NPOE), NaTPB, high relative molecular weight PVC, and tetra-hydrofuran (THF) were obtained from Fluka, or Merck and used as received. 0.0558 gr of AT was dissolved in 1.0 mL MeOH and 0.5 mL of HCl 0.01 M to form the AT(HCl)_2_ and then diluted to 10 mL by adding double-distilled water. Then the ion pair was prepared from aqueous medium by adding 5.0 mL of saturated NaTPB solution to 10 mL of10^-2^M of AT solution. The resulting ionpair precipitate was filtered, washed thoroughly with distilled water and dried under N_2_ atmosphere at room temperature. Stock solution of AT (5586000 ng mL^-1^) was prepared by dissolving an appropriate amount of the AT in MeOH-water (20-80%). Other dilute solutions (0.00558–5586 μg mL^-1^) were prepared by serial dilution and both the pH and ionic strength were kept constant at 5 and 0.01M, respectively. 

*Apparatus*

Potentiometric measurements were carried out at 25 ± 0*.*1 °C on a multichannel digital potentiometer pH/ion analyzer (model pH 162). The membranes were equilibrated for 1 day in 10^-3^M AT solution and the potentials were measured using PVC matrix membranes in conjunction with Ag/AgCl electrodes by setting up following electrochemical cell assembly:

The FT-IR measurement was carried out using Shimadzu (IR Prestige-21). The elemental analysis was performed using Heraeus CHN elemental Analyzer. The pH values of solutions were adjusted employing a Metrohm model 827 using a combined glass electrode. The chromatographic analysis was carried out according to our previous work (22) using a KNAURE instrument equipped with a power supplier and a UV detector. The HPLC was controlled by EZ-Chrome Elite software. The separation was performed on a Eurospher 100-5C18 column (240 × 4.0 mm i.d.). The mobile phase, consisted of MeOH–acetonitrile–0.05 M phosphate buffer (pH 3.0, 40:35:25 V/V%). The column was placed at an oven at 40 °C. The flow rate was 1.0 mL min–1, and the detection wavelength was 237 nm. The injection volume was 20 μL and the run time was 7.0 min.

*Electrode preparation*

The membrane composition was studied by varying the percentages (w/w %) of the ion pair, polyvinyl chloride (PVC) and plasticizer, until the optimum composition that exhibits the best performance characteristics was obtained (35- 37). The membranes were prepared by dissolving the required amount of the AT-(TPB)2, PVC and TOP, in 2.0 mL of THF. The solution mixture was poured into a 5.0 cm Petri dish and left to dry in air. To obtain the uniform membrane thickness, the amount of THF was kept constant in all preparations, and its evaporation was fixed for 4 h. A 15 mm diameter disk was cut out from the prepared membrane and glued using PVC–THF paste to the polished end of a plastic tube. The electrode body was filled with internal solution. The electrode is preconditioned by soaking in 558.6 μg mL^-1^ of AT solution. 

*Selectivity*

The potentiometric selectivity coefficients (*Kpot A.B ) *of the proposed sensor towards different substances were determined according to previous reported work (38) by a separate solution method using the following equation (I) (39):

$-\log(k_{A.B}^{\mathrm{pot}})=\frac{E_{1-E_{2}}}{2.303\frac{\mathrm{RT}}{Z_{A}F}}+(1-\frac{Z_{A}}{Z_{B}})\log a_{A}$          (I)

 where $K_{\mathrm{A.B}}^{\mathrm{POT}}$is the potentiometric selectivity coefficient, E_1_ is the potential measured in 10^-3^ M AT solution, E_2_ is the potential measured in 10^-3^ M interfering solution, ZA and ZB are the charges of AT and interfering ion, respectively, a_A_ is the activity of the drug and 2.303 $\frac{\mathrm{RT}}{\mathrm{ZAF}}$represents the calibration slope of the investigated sensor (mV/concentration decade). 

*Construction of calibration graph*

The conventional plastic membrane electrode was immersed in solutions of different concentrations of AT covering the concentration range 0.0055–5586 μg mL^-1^ of AT. The cell potential was recorded for each solution with constant stirring at 25 ± 0.1 °C and plotted against *log*[AT]. The slope of the calibration graph was calculated using Nernestain equation (II):

E=E1SE0+ 2.303 RTZF logAT             (II)

Where *R *is the gas constant, *F *is the Faraday equivalent and *z *is the charge of the analyte. The term EISE 0is a constant which is the sumof all invariants in the system. 

*Analysis of AT in lipitor, amostat, and biotor plus tablets*

The tablet sample preparations were performed in accordance of work of Hussein et al., (40). A homogenized powder was prepared from five accurately weighed of each Lipitor (10, 20, 40 mg) –contains atorvastatin (10, 20, 40 mg), Amostat (5/20, mg) contains–amlodipine/ atorvastatin (5/20 mg), and Biotor Plus (5/20 mg) contains–amlodipine/atorvastatin (5/20 mg) tablets. An appropriate amount of each tablet powder was transferred into a 10 mL Erlenmeyer flask. Dissolution of the drug was assisted by means of a magnetic stirrer and by addition of 2.0 mL of MeOH. The mixture was then filtered and made up to the mark with acetate buffer (pH = 5.0, 0.01 M) in a 50 mL volumetric flask. In accordance of the label for each tablet (10, 20, 40 mg AT per tablet, and5/20 mg amlodipine/ atorvastatinper tablet), the amount of AT should be 55860 ng mL^1^M in volumetric flask. The AT content was determined by the proposed membrane-selective electrode, using the calibration method. 

## Results and Discussions

*The proposed ion pair structure of AT-TPB*

**Figure 1:**
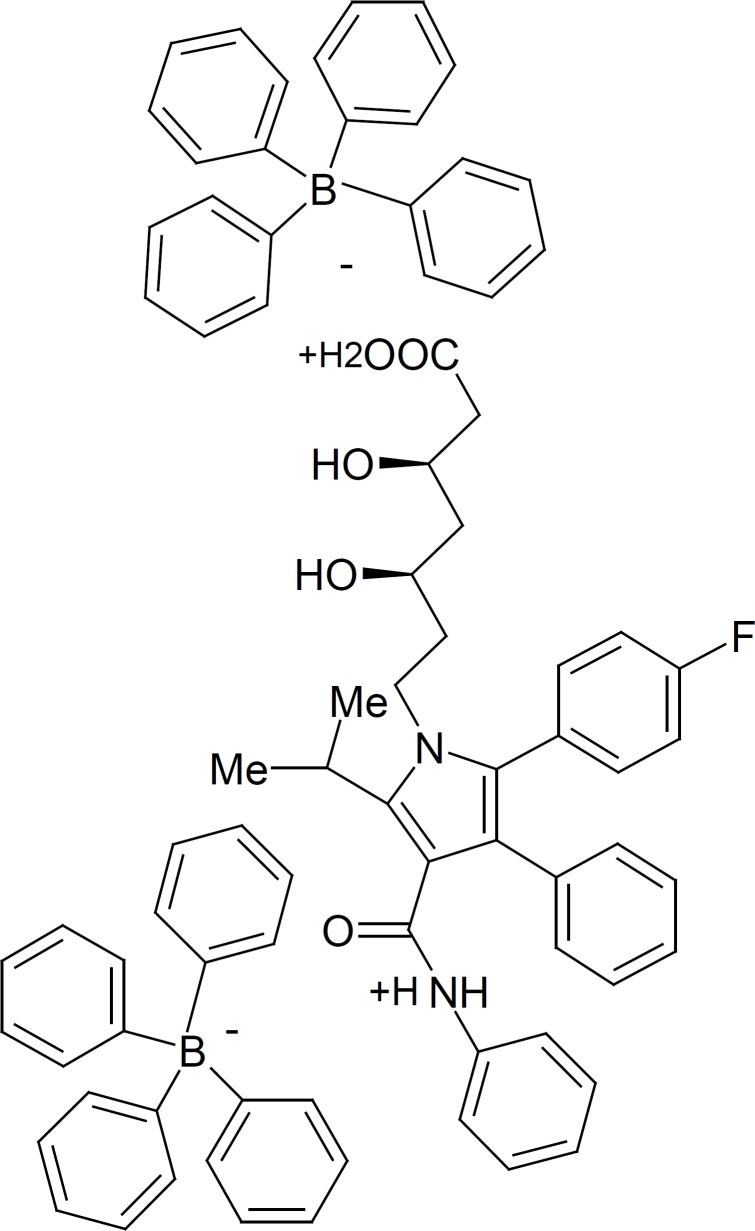
Suggested structure of atorvastatin-tetraphenylborate (AT-(TPB)_2_) ionpair

AT is a white to off–white crystalline powder with pK_a_= 4.46. It is insoluble in aqueous solutions with pH ≤ 4.0, while it is very slightly soluble in distilled water and freely soluble in methanol. For preparation of ionpair we found out that the AT is soluble in mixture of MeOH-0.01 M HCl solution (66:33%). The AT can behave as cation in acidic medium, due to presence of amid and carboxylic moiety (41). The composition of the precipitate was investigated by elemental analysis. The calculated percentage of C, H, and N for the AT-(TPB)_2_ (with mole ratio 1:2 was 81.16%, 6.42% and 2.34%, while the found percentages amounted to 80.5%, 6.21%, 2.26%, respectively. The results indicated the formation of a 1:2 ion pair (AT:TPB). Also the FT-IR measurements revealed that the –CO–NH– and –COOH moiety can be protonated and formed the ion pair with NaTPB. According to the calculated elemental analysis data and the FT-IR measurements for the ion-associate complex we proposed the follow structure for resulted 1:2 AT-(TPB)_2_ ionpair (Figure 1). 

*The evaluation of proposed ionpair as ionophor in PVC membrane*

**Figure 2:**
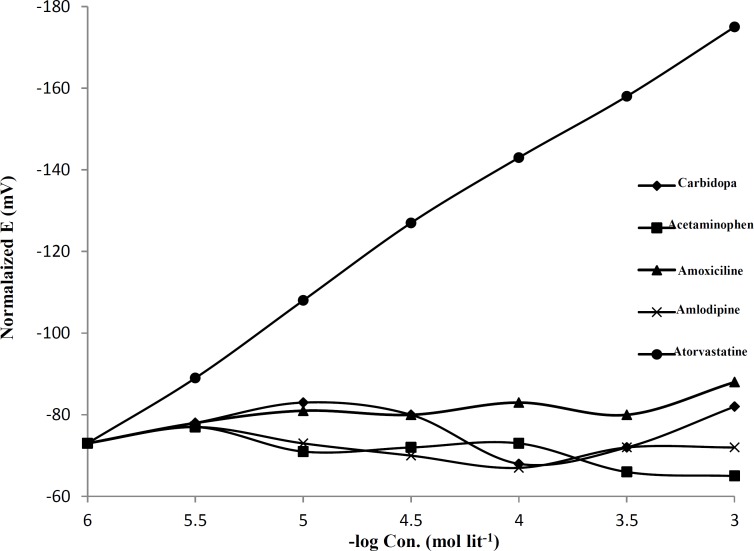
Electrode response for AT prepared with AT-(TPB)_2_ ionpair as ionophore, the membrane contain of PVC:NaTPB:ionopher: TOP with 35:1.0:1.5:65%, respectively, the pH=4.0

In the next experiments AT-(TPB)_2_ was used as an ionophor in construction of PVC-membrane electrode for AT. The potential response of constructed electrode for AT and several drugs are shown in Figure 2. As it is seen, except for the AT, for all other drugs, the slopes of the corresponding potential-*pM *plots are much lower than the expected Nernstian slope of 30 mV per decade for the 1:2 AT-(TPB)_2_ ion pair (42). 

*Investigation of membrane composition*

The proposed membrane sensor generates stable potential response in aqueous solutions containing AT after conditioning for 24 h in a 558.6 μg mL^-1^AT solution. Table 1 shows the data obtained with membranes having various ratios of different constituents. It is well known that some important features of the PVC-based membranes, such as the nature and amount of ionophor, the properties of the plasticizer, the plasticizer/PVC ratio and, especially, the amount of additive used, significantly influence the sensitivity and selectivity of the ion-selective electrodes (35, 36, 42). From the data, TOP was found to be the optimum available plasticizer for the PVC membrane sensor. It plasticizes the membrane, dissolves the ion-association complex and adjusts both of the membranes permittivity and ion-exchanger sites mobility to give highest possible selectivity and sensitivity (43). Other plasticizers such as TBP, NPOE, DBP and DOP failed in dissolving the ionpair and thus gave noisy responses. As can be seen from Table 1, by increasing amount of ion pair in membranes, the slopes were increased. Using 4% of AT-(TPB)_2_ and 65% of TOP in the membrane electrode displays Nernstian slope towards AT (membrane NO.9). As it is observed, (membrane NO. 12) the response of electrode to AT is affected only by the ionpair. Also, the sensitivity of the electrode in the absence of additive is poor (no.4 with a slope 27.2 mV/decade). Addition of additives (NaTPB) improves the sensitivity of the AT sensor. Table 1 revealed that addition of NaTPB as a suitable additive to the membrane increases the sensitivity of the sensor from 27.2 (NO.4) to 30.1 mV/decade (NO. 9). It is well established that the presence of lipophilic negatively charged additives improves the potentiometric behavior of certain selective electrodes not only by reducing the ohmic resistance and improving the response behavior and selectivity, but also, in cases where the extraction capability of the ionophor is poor, by enhancing the sensitivity of the membrane electrode (44). However, the best performance was obtained with membrane NO.9. The optimum response of the electrode was tested after conditioning for different periods of time in 558.6 μg mL^-1^ AT. The slope obtained using 24 h of conditioning was closer to the theoretical slope expected on the basis of the Nernstian equation. Longer conditioning times produced no further improvements in the response. The proposed PVC membrane electrode was used over a period of 1.0 month without any significant change in potential. During these times, the detection limit and the slope of the electrode remained almost constant. Subsequently, the electrochemical behavior of the electrode gradually deteriorated. This would be due to aging effect and leaching of the ion pair, NaTPB and the solvent mediator from the membrane into the solution by time.

*The effect of pH on electrode response*

**Figure 3:**
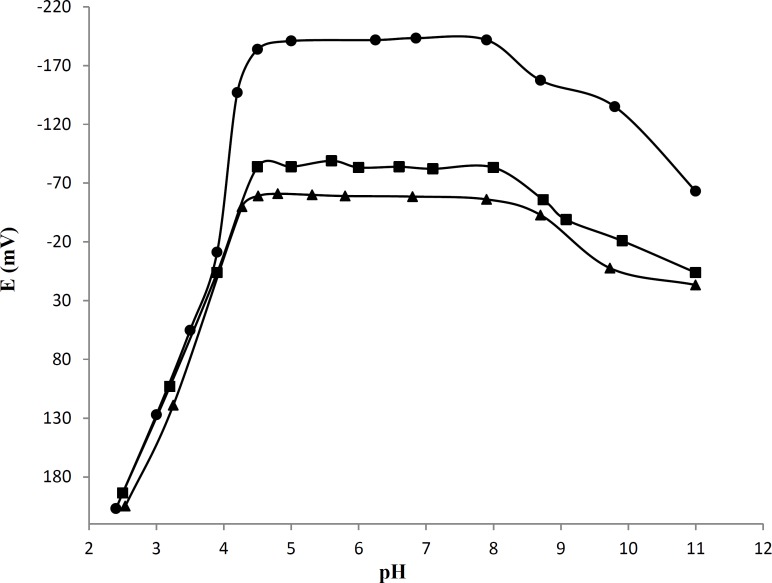
Effect of pH of the test solution on the electrode potential reading, (●) 2234, (¡) 1.4and (▲) 0.14 μg mL^-1^AT solutions, the composition of membrane was like to the membrane NO.9

The pH dependence of the electrode potential was tested over the pH range 2.5-11 for 2234, 1.4 and 0.14 μg mL^-1^ AT solutions. The pH was adjusted with NaOH or HCl solution. The potential was independent of pH in the range 4.5-8for three concentrations (Figure 3). In acidic pH the precipitation of AT was observed and the potential of the electrode moved to negative potentials with increasing in pH of the working solution. Over the pH 8.0, the potential decayed. In addition, the potentials displayed by the electrode were noisy as the ionpair may decompose and leaked in to the solution.

**Table 1:** Optimization of membrane ingredients

**NO**	**Percentage(W/W) of various components in membrane**									**L.R (μg mL** ^-1^ **)**	**RT (s)**
	**PVC**	**AT-(TPB)**_2_	**NaTPB**	**NPOE**	**DOP**	**TBP**	**TOP**	**DBP**	**slope**		
1	33.0	1.0	-	66.0	-	-	-		39.5	16.76 - 5586	20
2	33.0	1.0	-	-	66.0	-	-		22.2	223.4 – 5586	35
3	33.0	1.0	-	-	-	66.0	-		19.3	223.4 – 5586	40
4	33.0	1.0	-	-	-	-	66.0		27.2	3.9 – 5586	18
5	33.0	1.0	-	-	-	-	-	66.0	20.5	28 – 5586	25
6	33.5	1.0	0.5	-	-	-	65		28.5	0.44– 5586	18
7	35	1.0	1.5	-	-	-	65		27.9	2.8 – 5586	18
8	36.0	0.5	2.0	-	-	-	62		20.1	5.6 – 5586	20
9	30.0	4.0	1.0	-	-	-	65.0		30.1	0.09 – 5586	10
10	30.0	3.0	0.0	-	-	-	67.0		28.8	0.11 – 5586	12
11	30.0	3.0	1.0	-	-	-	66.0		29.4	0.11 – 5586	10
12	30	0.0	1.0	-	-	-	69		12	1117 – 5586	50

*Effect of temperature of the test solution*

**Figure 4:**
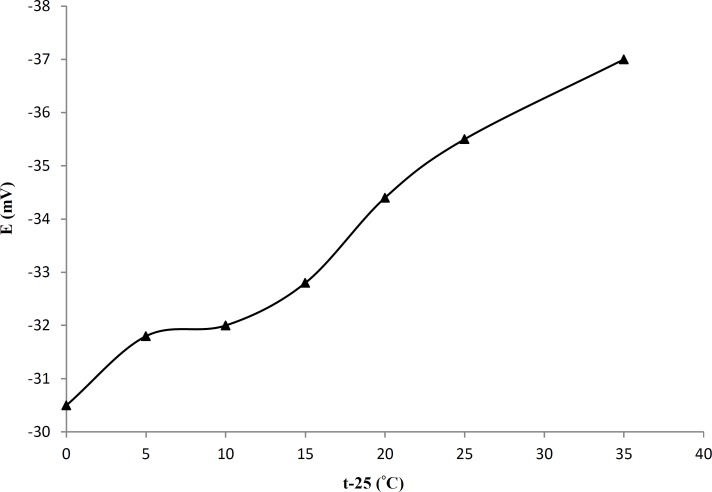
Variation of ˚*E*_elec_ with temperature

Calibration plot (*E *versus *pAT*) was constructed in the test solution temperature range 25–60 °C for proposed membrane. For the determination of the isothermal coefficients (*dE°/dt*) of the electrode, the standard electrode potentials (*E°*) of the cell at different temperatures were obtained from the calibration plots as the intercepts at *pAT *= 0. The slope, usable concentration range, and the standard potential (*E°*) of the electrode at each temperature are given in Table 2. For the determination of the isothermal coefficient (*dE°/dt*) of the cell, the standard electrode potentials *E°*, is plotted versus t-25 (Figure 4), where *t *is the temperature of the test solution (°C). A straight-line plot is obtained according to the following equation (III):

*E°= E°(25) + (dE°/dt)(t-25) *(III)

The slope of the straight line obtained represents the isothermal coefficient of 0.00018 V/°C for proposed electrode (45). This low value of isothermal temperature coefficient revealed that the electrode has good thermal stability within the studied temperature range (25-60 °C).

**Table 2:** Performance characteristics of proposed electrode at different temperatures

**Temperature (** **°** **C)**	**Slope (mV/decade)**	**Usable concentration range (pAT)**	**E** **° ** **(mV)**
25	30.1	5-2	-32.5
30	30.1	5-2	-32.8
35	30	5-2	-32
40	30	5-2	-32.8
45	29.8	4.8-2	-34.4
50	29.5	4.8-2	-35.5
60	29	4.5-2	-37

*Effect of internal reference solution*

The influence of the concentration of the internal solution was investigated for different concentrations of the AT solutions (55.8, 558.6, and 5586 μg mL^-1^). It was found that, by changing the concentration of the internal reference solution, the slope of the Nernstian plots remains nearly constant. Therefore, 558.6 μg mL^-1^concentration of the reference solution was quite appropriate for smooth functioning of the proposed sensor. 

*Response time*

**Figure 5:**
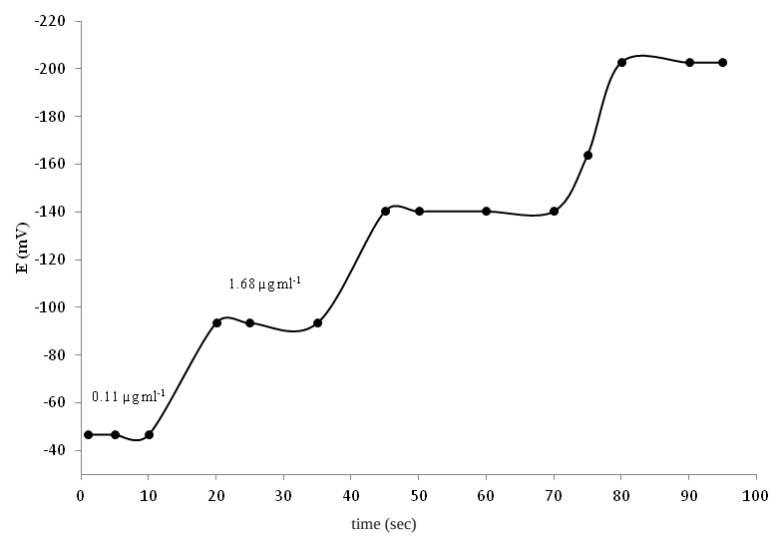
Dynamic response of the PVC membrane electrode based on AT-(TPB)_2_ for step change in activities of AT

Response time is an important factor for any potentiometric sensor. In fact response time is the time needed for the electrode to reach a stable. In this study, response time was recorded by changing the AT activity; over an activity range of 0.111-5586 μg mL^-1^. The resulting data depicted in Figure 5, show that the time required to achieve a steady potential after successive immersion of a series of AT drugis ≤10 s. 

*Selectivity*

The selectivity of the membrane in the presence of some inorganic cations, co-formulated drug (Amlodipine), other statin drugs, was investigated. The selectivity coefficients of electrode which determined by the separate solution method using Eq. (I) are presented in Table 3. The *−log(*$K_{\mathrm{A.B}}^{\mathrm{pot}}=\pi r^{2}$*) *values of the electrode show a high selectivity of the sensor for the AT. The selectivity of membrane is mainly dependence to how much matching is present between the locations of the lipophilic sites in the two competing species in the bathing solution side and those present in the receptor of the ion exchanger. The inorganic cations do not interfere owing to the differences in ionic size. In the case of other statin drugs, the high selectivity is mainly attributed to the difference in polarity and lipophilic character of their molecules relative to AT. 

**Table 3:** Selectivity coefficients *−log(*$K_{\mathrm{A.B}}^{\mathrm{pot}}$*) *of various interference with the AT-selective electrode

**Interfering compounds**	***−log(*** $K_{\mathrm{A.B}}^{\mathrm{pot}}$ ***)***	**Interfering compounds**	***−log(*** $K_{\mathrm{A.B}}^{\mathrm{pot}}$ ***)***
Carbidopa	4.6	Na+	4.5
Acetaminophen	3.5	K+	4.8
Amoxicillin	3.6	NH4+	4.4
Amlodipine	4.2	Cd2+	4.2
Levodopa	4.7	Cu2+	2.8
Minoxidil	3.5	Ag+	3.5
Hydrocortisone	4.1	Mg2+	4.1
Levostatine	3.8	Fe2+	4.2
Ca2+	3.4	Fe3+	3.1

*Effect of MeOH on membrane performance*

As the AT is insoluble in aqueous solutions with pH ≤ 4.0 and is very slightly soluble in distilled water, therefore, the sample preparation was performed in 20:80 %V/V of MeOH: acetate buffer, (see section 2.6). Therefore, the effect of MeOH on performance of the membrane was also studied in partially non-aqueous medium using water–methanol mixture. The membrane worked satisfactorily up to a maximum 20% (v/v) content of methanol. In these mixtures, the working concentration range and slope remained unaltered, however, above a 20% non-aqueous content the slope decreased appreciably and reliable measurements could not be obtained. Also, the membranes were destroyed due to leaching of the ionophore from the PVC matrix.

*Calibration curve, linearity range, detection limit, and reproducibility*

**Figure 6:**
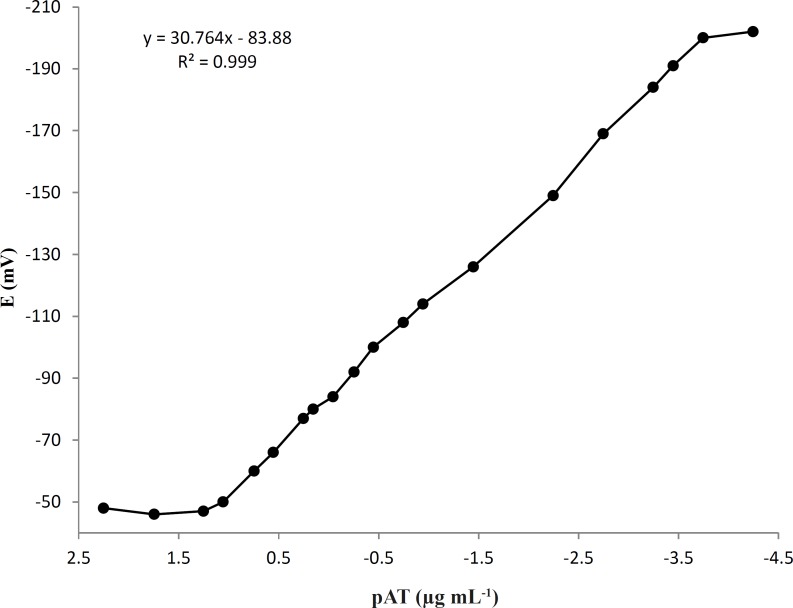
Calibration curve of AT (μg mL^-1^) obtained with proposed electrode at optimum membrane composition and measurement conditions

The choice of optimum membrane composition and measurement conditions must be resulted inacceptable calibration curve and a high degree of linearity over a usable range of concentrations. The best performance was exhibited by membrane containing the PVC:AT-(TPB)_2_: NaTPB: TOP in the ratio 30: 4.0: 1.0: 65, respectively. This membrane showed a nearly Nernestian response with slope of 30.1 ± 0.1 mV/decade and a linear concentration range 0.09–5586 μg mL^-1^ (Figure 6). The detection limit, defined as the cross section of the two extrapolated linear segments of the calibration curve, was0.056 μg mL^-1^. Five replicate determinations at three different concentration levels (0.5, 50, 5000 ng mL^-1^) were carried out using the proposed electrode to test the precision of the method. The relative standard deviations (RSD) were found 2.18%, 1.98, and 1.54%, respectively, indicating reasonable repeatability and reproducibility of the selected method. 

*Analytical applications*

The investigated electrode was proved to be useful in the potentiometric determination of AT in different tablets such as Lipitor (10, 20, 40 mg)–contains atorvastatin (10, 20, 40 mg), Amostat (5/20 mg)–contain amlodipine/atorvastatin (5/20 mg), and Biotor Plus (5/20 mg)–contain amlodipine/atorvastatin (5/20 mg). Collective results are given in Table 4. From the results, it is evident that the present electrode is very useful as a potentiometric sensor for the determination of AT in pharmaceutical preparations. It must be mentioned that the electrode should be calibrated every day before measurements. If any deterioration in the slope is observed, the electrode must be replaced with another new one. To compare the proposed method to a reported method, AT in three Amostat, Biotor Plusand Lipitor tablets were assayed by HPLC method. Statistical comparison of the results of the proposed and reported methods (Table 4) was performed with regard to accuracy and precision using the t- and F-ratio tests. At 95% confidence level, the calculated *t- *and *F-*values did not exceed the critical values, indicating that there is no significant difference between the proposed method and HPLC method with regard to accuracy and precision.

**Table 4:** determination of AT content in five types of tablets

**Tablet Name**	**Proposed sensor (n=4)**			**HPLC (n=4)**
	**Add (mg/tablet)**	**Found (mg/tablet) ±SD**	**Recovery%**	**Found (mg/tablet) ±SD**
Lipitor (10)	0.0	9.85±0.18	98.5	9.94±0.12
	2.0	11.83±0.24	98.5	12.03±0.18
	5.0	14.80±0.25	98.6	15.12±0.21
^a^t- test (95%; 3.18) aF-test(95% ; 9.28)		0.83 2.25		
Lipitor (20)	0.0	19.95±0.39	99.7	20.1±0.25
	2.0	22.05±0.45	100.0	22.14±0.24
	5.0	25.14±0.40	100.6	24.97±0.34
^a^t- test (95%; 3.18) aF-test(95% ; 9.28)		0.65 2.43		
Lipitor (40)	0.0	40.2±0.77	100.5	39.87±0.41
	2.0	41.89±0.70	99.7	42.13±0.50
	5.0	44.8±0.80	97.7	45.10±0.65
^a^t- test (95%; 3.18) aF-test(95% ; 9.28)		0.76 3.53		
Amostat (5/20)	0.0	19.75±0.35	98.7	19.89±0.24
	2.0	22.1±0.49	100.4	21.98±0.27
	5.0	24.85±0.41	99.4	25.12±0.31
^a^t- test (95%; 3.18) aF-test(95% ; 9.28)		0.66 2.13		
Biotor Plus (5/20)	0.0	19.9±0.37	99.5	20.13±0.27
	2.0	21.87±0.47	22.4	22.05±0.32
	5.0	24.75±0.41	99.0	25.09±0.28
^a^t- test (95%; 3.18) aF-test(95% ; 9.28)		3.10 1.88		

## Conclusion

We founded that, this plasticized PVC membrane containing AT-(TPB)_2_ has been shown good sensitivity, reproducibility, selectivity and better life time. Also, it is very easy to prepare and has a wide linear dynamic range and low detection limit. The electrode works in a pH range of 4.5– 8, which makes it useful for measurements of AT in neutral and physiological pH. High selectivity, low detection limit, and rapid response make this electrode suitable for measuring the concentration of AT in pharmaceutical preparations, without any preconcentration or pretreatment steps. Table 5 compared the performance characteristics of the proposed electrode with other previously reported works for analysis of AT. Although, this work has higher limit of detection (LOD) and lower linear range (L.R) in comparison with other methods such as LC-MS or HPLC-UV, but it is more selective, easy to performance, economic, simple and does not need sample preparation.

**Table 5:** the comparison of proposed sensor with other analytical methods for analysis of AT

**Method Name**	**Sample preparation**	**L.R (μg mL** ^-1^ **)**	**LOD (μg mL** ^-1^ **)**	**Ref.**
Derivative Spectrophotometry	--	10–35	1.75	[46]
Spectrofluorimetry	--	1.5–4.0	0.012	[47]
LC-MS/MS	Protein Precipitation	0.0005–0.15	--	[48]
capillary electrophoresis	--	1.0–100	60	[18]
LC-MS	Solid phase extraction	0.0002–0.03	0.00006	[49]
HPLC-UV	Liquid-liquid-liquid microextraction	0.001–0.5	0.0004	[50]
Proposed sensor	--	0.09–5586	0.056	
